# Synthesis and Fungicidal Activity of Novel Chloro-Containing 1-Aryl-3-oxypyrazoles with an Oximino Ester or Oximino Amide Moiety

**DOI:** 10.3390/molecules19068140

**Published:** 2014-06-17

**Authors:** Yuanyuan Liu, Yi Li, Nanqing Chen, Kunzhi Lv, Chao Zhou, Xiaohui Xiong, Fangshi Li

**Affiliations:** 1 Department of Chemical and Pharmaceutical Engineering, Southeast University ChengXian College, Nanjing 210088, China; 2 College of Food Science and Light Industry, Nanjing Tech University, Nanjing 211816, China; 3 College of Science, Nanjing Tech University, Nanjing 211816, China

**Keywords:** synthesis, fungicidal activity, chloro-containing 1-aryl-3-oxypyrazoles, structure-activity relationships

## Abstract

Six novel chloro-containing 1-aryl-3-oxypyrazoles **TMa**–**TMf** with an oximino ester or an oximino amide moiety were prepared by the reaction of 1-aryl-1*H*-pyrazol-3-ols with benzyl bromide. Their structures were characterized by ^1^H-NMR, ^13^C-NMR, IR, MS, and elemental analysis. A preliminary *in vitro* bioassay indicated that compounds **TMa**, **TMe** and **TMf** displayed excellent fungicidal activity against *Rhizoctonia solani* and could be used as potential lead compounds for further development of novel fungicides.

## 1. Introduction

**Figure 1:**
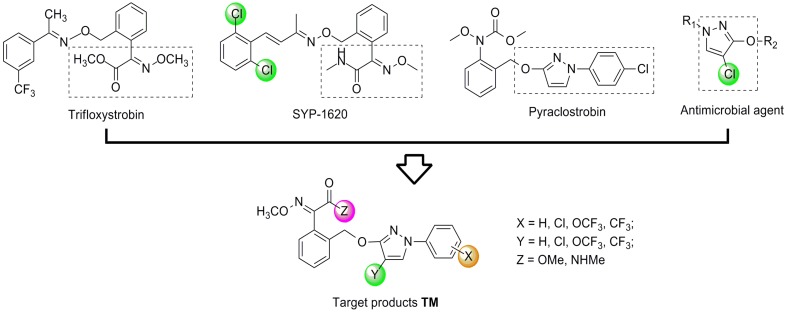
Design strategy for the target products **TM**.

Since the discovery of the strobilurin fungicide azoxystrobin by Syngenta scientists [1], this novel fungicide class has occupied an important position in the agrochemical market due to its lower mammalian toxicity, wider spectrum, and higher bioactivity [2,3,4]. Generally, the chemical structure of strobilurin fungicides can be divided into three parts: (A) an (*E*)-*β*-methyl methoxyiminoacetate or an isosteric (*E*)-*β*-methyl methoxyacrylate moiety as pharmacophore; (B) an aromatic bridge moiety; and (C) a side chain. Previous researchers have mainly focused on maintaining the pharmacophore but changing the side chain, and some new strobilurins such as trifloxystrobin, SYP-1620 and pyraclostrobin (Figure 1) have been commercialized [5,6,7,8]. Our group has also devoted considerable effort to the development of novel fungicides. Bioactive (alkyl)oxyacetate, thiazolidine-2-thione, (*O*-acetyl)gluco- pyranosyl or benzoyl moieties were introduced into the 1-aryl-3-oxypyrazole structure of pyraclostrobin to replace its methoxycarbamate pharmacophore which could not be prepared in an environmentally friendly way, and some novel aryloxypyrazoles with good fungicidal activity were reported [9,10,11].

Furthermore, several biological studies have also indicated the 4-chloro-3-oxypyrazole moiety as a bioactive structure, and side chain modifications such as introduction of chlorine can indeed lead to a better fungicidal efficacy [12,13]. In continuation of our studies on fungicidal aryloxypyrazoles, the chlorine and oximino ester or oximino amide pharmacophore of strobilurins were introduced into the 1-aryl-3-oxypyrazole structure of pyraclostrobin according to the principle of active parts combination, and new target products **TM** were designed (Figure 1).

In this paper, the synthesis of six novel chloro-containing 1-aryl-3-oxypyrazoles **TMa**–**TMf** with an oximino ester or an oximino amide moiety is reported. Meanwhile, their fungicidal activity has been investigated with the aim of understanding the structure-activity relationships and developing novel fungicides. Preliminary *in vitro* bioassay data indicated that some compounds showed excellent fungicidal activity against *Rhizoctonia solani*, especially **TMa**, **TMe** and **TMf**, which displayed a higher activity than pyraclostrobin at a dosage of 0.1 μg/mL.

## 2. Results and Discussion

### 2.1. Chemistry

**Scheme 1:**
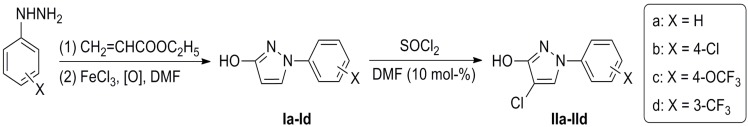
Synthesis of intermediate 4-chloro-1-aryl-1*H*-pyrazol-3-ols **IIa**–**IId**.

**Scheme 2:**
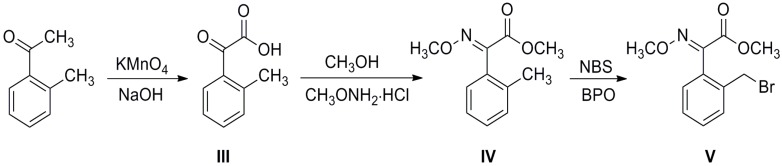
Synthesis of intermediate benzyl bromide **V**.

Intermediate 4-chloro-1-aryl-1*H*-pyrazol-3-ols **IIa**–**IId** were synthesized from arylhydrazines via addition-cyclization, oxidation and chlorination (Scheme 1) [14,15]. A previous report by Li and Yang described that the benzyl bromide (*E*)-methyl 2-(2-(bromomethyl)phenyl)-2-(methoxyimino)acetate **V** could be prepared from 1-(*o*-tolyl)ethanone *via* a serial of reactions including oxidation, esterification, oximation and bromination [16]. However, due to the fact the oxidation was carried out under heterogeneous conditions, the yield was low, so in our procedure, we optimized the reaction conditions, and the oxidation was carried out by phase-transfer catalysis with tetrabutylammonium bromide (Bu_4_N^+^Br^−^) under alkaline conditions, which gave 2-oxo-2-(*o*-tolyl)acetic acid **III** in 80% yield (Scheme 2). In the following step, the esterification of **III** was carried out in methanol, using concentrated sulfuric acid as catalyst. The compound obtained was directly used for oximation without further purification. The bromination of oximino ester **IV** was carried out in a molar ratio of **IV **to *N*-bromosuccinimide (NBS) 1:1.1 equiv. in CCl_4_ as solvent, and with benzoyl peroxide (BPO) as catalyst.

**Scheme 3:**
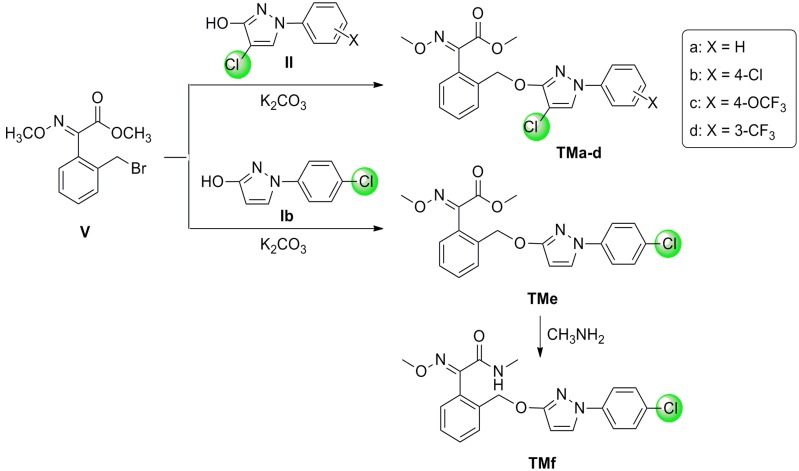
Synthesis of target products **TMa**–**TMf**.

In our previous studies, we have prepared several ethyl 2-(1,5-diaryl-1*H*-pyrazol-3-yloxy)acetates by the substitution reaction of 1,5-diaryl-1*H*-pyrazol-3-ols with ethyl 2-bromoacetate in acetone, using potassium carbonate (K_2_CO_3_) as acid-binding agent [11]. Motivated by this reaction, in our procedure, hydroxypyrazoles **Ib** and **IIa**–**IId** were allowed to react with benzyl bromide **V** in a 1:1.1 molar equiv. ratio in boiling acetone in the presence of K_2_CO_3_, which afforded the target products **TMa**–**TMe** in 80%–82% yield as sole isolable products (Scheme 3). The reaction process for **TMf** was carried out in a molar ratio of **TMe** to methylamine in anhydrous methanol (30%) 1:3 equiv. at reflux temperature until **TMe** was completely consumed as judged by TLC. The crude product was purified *via* column chromatography to give the pure **TMf** in 90% isolated yield (Scheme 3).

The structures of **TMa**–**TMf** were confirmed by their NMR spectra. In the ^1^H-NMR spectra, as a result of the deshielding effect of aryl and chloro groups, the CH of the pyrazole ring in **TMa**–**TMd** appeared at low field (*δ* 7.68–7.81 ppm) as a singlet, whereas the corresponding CH proton in **TMe**–**TMf** appeared at *δ* 7.65 ppm and *δ* 5.85 ppm, respectively, as two doublets with coupling constants around 2.5 Hz. The aromatic protons of **TMa**–**TMf** resonated in the range of *δ* 7.79–7.19 ppm in ^1^H-NMR spectra, and the ^13^C-NMR signals were observed around *δ* 146.6–114.3 ppm. The chemical shifts of MeO H-atoms in **TMa**–**TMe** appeared as two singlets around *δ* 4.06 ppm and *δ* 3.84 ppm, respectively, whereas one MeO proton signal in **TMf** was absent and replaced by the Me proton at *δ* 2.89 ppm. All compounds exhibited a carbonyl (C=O) ^13^C signal at the lowest field in the region of *δ* 163.9–163.3 ppm.

### 2.2. Fungicidal Activity

Products **TMa**–**TMf** were screened for fungicidal activity against *Rhizoctonia solani* at the dosages of 10, 1, and 0.1 μg/mL, respectively. As can be seen in Table 1 and Table 2, most compounds have good fungicidal activity at 10 μg/mL, especially **TMa**, **TMe** and **TMf**, with 100% antifungal activity. When the concentration was reduced to 0.1 μg/mL, **TMe**, **TMa** and **TMf** also had 99%, 83% and 71% inhibition rates, which were better than those of the reference compound pyraclostrobin. Compound **TMe** had an optimal EC_90_ lower than 0.1 μg/mL, which was better than **TMa** (EC_90_ = 0.20 μg/mL) and **TMf** (EC_90_ = 0.32 μg/mL). The 4-chloropyrazole containing a Cl on the phenyl ring (**TMb**) displayed higher activity than that with an electron-donating CF_3_O (**TMc**) or electron-withdrawing CF_3_ (**TMd**) group. According to the different positions of chlorine, the sequence of fungicidal activity is Cl-substituted phenyl ring > Cl-substituted pyrazole ring. For example, the structure only containing a Cl on the phenyl ring (**TMe**) showed better fungicidal activity than that on the pyrazole ring (**TMa**). However, with increasing number of the Cl groups, the fungicidal activity was decreased, as seen in the comparison of **TMa** and **TMe** *vs.* **TMb**, which might because the larger molecular volume was unfavourable for the intracellular uptake and transport in the fungus. In terms of the pharmacophore, the compound with an oximino ester moiety (**TMe**) had better fungicidal activity than that with an oximino amide (**TMf**) or a methoxycarbamate moiety (pyraclostrobin). The present work indicated that **TMa**, **TMe** and **TMf** could be used as potential lead compounds for further studies of novel fungicides.

**Table 1:** Antifungal activity of tested compounds (% inhibition).

Compounds	Structure	*Rhizoctonia solani* ^a^		
		10 μg/mL	1 μg/mL	0.1 μg/mL
**TMa**	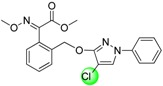	+++	+++	+++
**TMb**	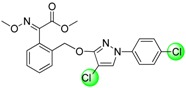	+++	+++	+
**TMe**	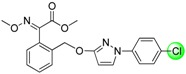	+++	+++	+++
**TMf**	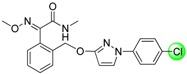	+++	+++	++
**TMc**	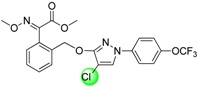	++	+	+
**TMd**	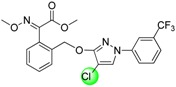	++	+++	++
Pyraclostrobin	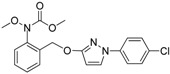	++	+	+

^a^ Activity is expressed in four categories: (−) < 20%, (+) 21%–50%, (++) 51%–80%, and (+++) 81%–100%.

**Table 2:** Antifungal activity and EC_90_ values of compounds **TMa**, **TMe** and **TMf**.

Compounds	concn (μg/mL) ^a^			EC_90_ (μg mL^−1^)
	10	1	0.1	
**TMa**	100.00	98.23	83.40	0.20
**TMe**	100.00	100.00	98.94	<0.1
**TMf**	100.00	94.70	71.40	0.32

^a^ 100.00 = total kill.

## 3. Experimental

### 3.1. General Information

Melting points were measured on an X-4 microscope electrothermal apparatus (Taike, Beijing, China) and were uncorrected. NMR spectra were obtained in CDCl_3_ or DMSO-d_6_ on a Brucker NMR spectrometer operating at 400 MHz for ^1^H and 100 MHz for ^13^C, with TMS as an internal standard. Elemental analyses were performed on a Flash EA-1112 elemental analyzer.

### 3.2. Synthesis and Characterization

Intermediates 1-aryl-1*H*-pyrazol-3-ols **Ia**–**Id** were prepared according to the reported method, and the spectral data matched those previously reported [14].

#### 3.2.1. General Procedure for the Synthesis of **IIa**–**IId**

A mixture of SOCl_2_ (5 mL), **Ia**–**Id** (1.0 mmol), and a catalytic amount of DMF (0.1 mmol) were heated under reflux for about 4 h (monitored by TLC), then excess SOCl_2_ was evaporated and H_2_O (200 mL) was added with good stirring. The precipitate that formed was filtered off, washed with H_2_O, and then purified by a silica-gel column chromatography (petroleum ether/EtOAc = 5:1) to afford **IIa**–**IId**.

*4-Chloro-1-phenyl-1*H*-pyrazol-3-ol* (**IIa**). Yield 80%; White solid; mp 183–184 °C; ^1^H-NMR (DMSO-d_6_) *δ* 11.00 (s, 1H, OH), 8.54 (s, 1H, CH), 7.69–7.21 (m, 5H, Ar-H). Anal. calcd for C_9_H_7_ClN_2_O: C 55.54, H 3.63, N 14.39; found C 55.65, H 3.62, N 14.34.

*4-Chloro-1-(4-chlorophenyl)-1*H*-pyrazol-3-ol* (**IIb**)*.* Yield 85%; White solid; mp 204–205 °C; ^1^H-NMR (CDCl_3_) *δ* 7.69 (s, 1H, CH), 7.46–7.39 (m, 4H, Ar-H). Anal. calcd for C_9_H_6_Cl_2_N_2_O: C 47.19, H 2.64, N 12.23; found C 47.12, H 2.64, N 12.26.

*4-Chloro-1-(4-(trifluoromethoxy)phenyl)-1*H*-pyrazol-3-ol* (**IIc**)*.* Yield 78%; White solid; mp 172–173 °C; ^1^H-NMR (CDCl_3_) *δ* 7.62 (s, 1H, CH), 7.40 (d, *J* = 8.8 Hz, 2H, Ar-H), 7.25 (d, *J* = 8.8 Hz, 2H, Ar-H). Anal. calcd for C_10_H_6_ClF_3_N_2_O_2_: C 43.11, H 2.17, N 10.05; found C 43.33, H 2.13, N 10.12.

*4-Chloro-1-(3-(trifluoromethyl)phenyl)-1*H*-pyrazol-3-ol* (**IId**)*.* Yield 76%; White solid; mp 210–211 °C; ^1^H-NMR (CDCl_3_) *δ* 7.74 (s, 1H, CH), 7.66–7.46 (m, 4H, Ar-H). Anal. calcd for C_10_H_6_ClF_3_N_2_O: C 45.73, H 2.30, N 10.67; found C 45.55, H 2.26, N 10.75.

#### 3.2.2. General Procedure for the Synthesis of **III**

A mixture of 1-(*o*-tolyl)ethanone (10 g, 74.6 mmol), H_2_O (150 mL), NaOH (2 g, 50 mmol) and Bu_4_N^+^Br^−^ (0.1 g, 0.295 mmol) was stirred at 0 °C, then KMnO_4_ (23 g, 0.146 mol) was added gradually. The mixture was stirred at 30 °C until the color of the solution remained unchanged. The black precipitate was filtered off, and the filtrate was neutralized by NaHSO_3_ solution, acidified with HCl (36.5%) and filtered. The filtrate was extracted with EtOAc, and then the solvent was removed under reduced pressure to afford 2-oxo-2-(*o*-tolyl)acetic acid (**III**). Yield 80%; White oil; ^1^H-NMR (CDCl_3_) *δ* 7.83–7.25 (m, 4H, Ar-H), 2.52 (s, 3H, CH_3_). Anal. calcd for C_9_H_8_O_3_: C 65.85, H 4.91; found C 65.62, H 4.93.

#### 3.2.3. General Procedure for the Synthesis of **IV**

A mixture of **III** (5 g, 0.03 mol), H_2_SO_4_ (1 mL), and MeOH (100 mL) was heated to reflux until the starting material had been completely consumed as judged by TLC. The mixture was neutralized by NaHCO_3_, and CH_3_ONH_2_·HCl (3 g, 0.036 mol) was added. The reaction was monitored by TLC, then excess MeOH was removed under reduced pressure. The residue was neutralized by NaHCO_3_, extracted by EtOAc, dried, filtered, and evaporated under reduced pressure. It was then purified by a silica-gel column chromatography (petroleum ether/EtOAc = 8:1) to afford (*E*)-methyl 2-(methoxy- imino)-2-(*o*-tolyl)acetate (**IV**). Yield 70%; White solid; mp 63–64 °C; ^1^H-NMR (CDCl_3_) *δ* 7.31–7.10 (m, 4H, Ar-H), 4.05 (s, 3H, OCH_3_), 3.87 (s, 3H, OCH_3_), 2.19 (s, 3H, CH_3_). Anal. calcd for C_11_H_13_NO_3_: C 63.76, H 6.32, N 6.76; found C 63.98, H 6.29, N 6.74.

#### 3.2.4. General Procedure for the Synthesis of **V**

To the solution of **IV** (5 g, 24.0 mmol) in CCl_4_ (150 mL) was added NBS (4.7 g, 26.4 mmol) and BPO (1 g, 4.13 mmol). The reaction mixture was heated to reflux until the starting material had been completely consumed as judged by TLC. The precipitate was filtered off, and the solvent was evaporated under reduced pressure. The residue was purified by a silica-gel column chromatography (petroleum ether/EtOAc = 15:1) to give (*E*)-methyl 2-(2-(bromomethyl)phenyl)-2-(methoxyimino)- acetate (**V**). Yield 85%; Yellow oil; ^1^H-NMR (CDCl_3_) *δ* 7.47-7.13 (m, 4H, Ar-H), 4.32 (s, 2H, CH_2_), 4.04 (s, 3H, OCH_3_), 3.85 (s, 3H, OCH_3_). Anal. calcd for C_11_H_12_BrNO_3_: C 46.18, H 4.23, N 4.90; found C 46.32, H 4.21, N 4.88.

#### 3.2.5. General Procedure for the Synthesis of **TMa**–**TMe**

To a solution of **Ib** and **IIa**–**IId** (1.0 mmol) in acetone (30 mL) was added K_2_CO_3_ (1.5 mmol). The mixture was refluxed for 15 min and **V** (1.05 mmol) was added slowly. The mixture was refluxed for about 4 h, then K_2_CO_3_ was filtered off. The solvent was evaporated under reduced pressure. The residue was purified by a silica-gel column chromatography (petroleum ether/EtOAc = 10:1) to afford products **TMa**–**TMe**.

*(E)-Methyl 2-(2-{[(4-chloro-1-phenyl-1*H*-pyrazol-3-yl)oxy]methyl}phenyl)-2-(methoxyimino)acetate* (**TMa**). Yield 80%; White solid; mp 104–105 °C; ^1^H-NMR (CDCl_3_) *δ* 7.73 (s, 1H, CH), 7.63 (d, *J* = 7.4 Hz, 1H, oximino-ArH), 7.53–7.19 (m, 8H, ArH), 5.25 (s, 2H, CH_2_), 4.04 (s, 3H, MeO), 3.84 (s, 3H, MeO). ^13^C-NMR (CDCl_3_) *δ* 163.3, 158.9, 149.4, 139.7, 134.8, 130.1, 129.5, 129.4, 128.9, 128.5, 128.1, 125.7, 125.6, 117.6, 98.3, 69.5, 63.9, 53.1. IR (KBr): 3147, 2949, 2895, 1734, 1598, 1557, 1511, 1463, 1438, 1406, 1356, 1323, 1223, 1122, 1063, 1012, 984, 947, 895, 862, 795, 756, 684. MS (ESI): calcd for [M+H]^+^ 400.11, [M+Na]^+^ 422.09; found [M+H]^+^ 400.11, [M+Na]^+^ 422.09. Anal. calcd for C_20_H_18_ClN_3_O_4_: C 60.08, H 4.54, N 10.51; found C 59.83, H 4.58, N 10.57.

*(E)-Methyl 2-(2-{[(4-chloro-1-(4-chlorophenyl)-1*H*-pyrazol-3-yl)oxy]methyl}phenyl)-2-(methoxyimino) acetate* (**TMb**)*.* Yield 82%; White solid; mp 147–148 °C; ^1^H-NMR (CDCl_3_) *δ* 7.68 (s, 1H, CH), 7.61 (d, *J* = 7.4 Hz, 1H, oximino-ArH), 7.44 (d, *J* = 8.0 Hz, 2H, N-ArH), 7.40 (d, *J* = 8.0 Hz, 2H, N-ArH), 7.33 (m, 2H, oximino-ArH), 7.21 (dd, *J* = 1.6, 7.4 Hz, 1H, oximino-ArH), 5.23 (s, 2H, CH_2_), 4.03 (s, 3H, MeO), 3.84 (s, 3H, MeO).^ 13^C-NMR (CDCl_3_) *δ* 163.3, 159.0, 149.4, 138.2, 134.7, 131.0, 130.1, 129.5, 128.9, 128.5, 128.2, 125.6, 118.6, 98.8, 69.5, 63.9, 53.1. IR (KBr): 3140, 2940, 1721, 1594, 1547, 1495, 1437, 1355, 1327, 1253, 1217, 1114, 1063, 1016, 959, 829, 785, 723, 689. MS (ESI): calcd for [M+H]^+^ 434.07, [M+Na]^+^ 456.05; found [M+H]^+^ 434.07, [M+Na]^+^ 456.05. Anal. calcd for C_20_H_17_Cl_2_N_3_O_4_: C 55.31, H 3.95, N 9.68; found C 55.52, H 3.91, N 9.62.

*(E)-Methyl 2-(2-{[(4-chloro-1-(4-(trifluoromethoxy)phenyl)-1*H*-pyrazol-3-yl)oxy]methyl}phenyl)-2- (methoxyimino)acetate* (**TMc**)*.* Yield 75%; White solid; mp 149–150 °C; ^1^H-NMR (CDCl_3_) *δ* 7.71 (s, 1H, CH), 7.62 (d, *J* = 7.2 Hz, 1H, oximino-ArH), 7.52 (d, *J* = 8.4 Hz, 2H, N-ArH), 7.42 (m, 2H, oximino-ArH), 7.25 (d, *J* = 8.4 Hz, 2H, N-ArH), 7.21 (dd, *J* = 1.6, 7.4 Hz, 1H, oximino-ArH), 5.24 (s, 2H, CH_2_), 4.04 (s, 3H, MeO), 3.84 (s, 3H, MeO).^ 13^C-NMR (CDCl_3_) *δ* 163.3, 159.2, 149.4, 146.6, 138.2, 134.7, 130.1, 129.5, 128.9, 128.5, 128.2, 125.7, 122.2, 118.6, 99.0, 69.6, 63.9, 53.0. IR (KBr):3144, 2946, 1722, 1607, 1558, 1507, 1441, 1393, 1370, 1298, 1261, 1208, 1146, 1115, 1063, 1017, 957, 842, 808, 759, 728, 685. MS (ESI): calcd for [M+H]^+^ 484.09, [M+Na]^+^ 506.07; found [M+H]^+^ 484.09, [M+Na]^+^ 506.06. Anal. calcd for C_21_H_17_ClF_3_N_3_O_5_: C 52.13, H 3.54, N 8.68; found C 52.35, H 3.60, N 8.61.

*(E)-Methyl 2-(2-{[(4-chloro-1-(3-(trifluoromethyl)phenyl)-1*H*-pyrazol-3-yl)oxy]methyl}phenyl)-2- (methoxyimino)acetate* (**TMd**)*.* Yield 83%; White solid; mp 119–120 °C; ^1^H-NMR (CDCl_3_) *δ* 7.81 (s, 1H, CH), 7.79 (s, 1H, N-ArH), 7.68 (d, *J* = 8.0 Hz, 1H, N-ArH), 7.62 (d, *J* = 7.4 Hz, 1H, oximino-ArH), 7.51 (t, *J* = 7.6 Hz, 1H, N-ArH), 7.42 (m, 3H, oximino-ArH), 7.21 (t, *J* = 7.6 Hz, 1H, N-ArH), 5.26 (s, 2H, CH_2_), 4.05 (s, 3H, MeO), 3.85 (s, 3H, MeO).^ 13^C-NMR (CDCl_3_) *δ* 163.3, 159.3, 149.4, 140.0, 134.5, 130.2, 130.1, 129.5, 129.0, 128.5, 128.2, 125.6, 122.1, 122.0, 120.1, 114.3, 99.6, 69.7, 63.9, 53.1. IR (KBr): 3140, 2946, 1730, 1598, 1562, 1518, 1458, 1406, 1358, 1328, 1306, 1226, 1169, 1122, 1067, 1014, 948, 889, 794, 758, 692. MS (ESI): calcd for [M+H]^+^ 468.09, [M+Na]^+^ 490.08; found [M+H]^+^ 468.10, [M+Na]^+^ 490.08. Anal. calcd for C_21_H_17_ClF_3_N_3_O_4_: C 53.91, H 3.66, N 8.98; found C 54.12, H 3.60, N 8.92.

*(E)-Methyl 2-(2-(((1-(4-chlorophenyl)-1*H*-pyrazol-3-yl)oxy)methyl)phenyl)-2-(methoxyimino)acetate* (**TMe**)*.* Yield 82%; White solid; mp 131–132 °C; ^1^H-NMR (CDCl_3_) *δ* 7.66 (d, *J* = 2.4 Hz, 1H, CH), 7.60 (d, *J* = 7.2 Hz, 1H, oximino-ArH), 7.52 (d, *J* = 9.0 Hz, 2H, N-ArH), 7.42 (m, 2H, oximino-ArH), 7.36 (d, *J* = 9.0 Hz, 2H, N-ArH), 7.20 (dd, *J* = 1.8, 7.2 Hz, 1H, oximino-ArH), 5.85 (d, *J* = 2.4 Hz, 1H, CH), 5.18 (s, 2H, CH_2_), 4.08 (s, 3H, OCH_3_), 3.84 (s, 3H, OCH_3_). ^13^C-NMR (CDCl_3_) *δ* 163.7, 163.0, 149.1, 138.3, 134.8, 130.1, 129.4, 129.1, 129.0, 128.1, 127.5, 127.3, 119.2, 118.5, 94.2, 68.7, 63.4, 52.6. IR (KBr): 3142, 2997, 2945, 1740, 1589, 1542, 1475, 1357, 1306, 1225, 1069, 1016, 978, 935, 895, 845, 784, 749. MS (ESI): calcd for [M+H]^+^ 400.11, [M+Na]^+^ 422.09; found [M+H]^+^ 400.10, [M+Na]^+^ 422.09. Anal. calcd for C_20_H_18_ClN_3_O_4_: C 60.08, H 4.54, N 10.51; found C 59.85, H 4.59, N 10.58.

#### 3.2.6. General Procedure for the Synthesis of **TMf**

Compound **TMe **(0.4 g, 1.0 mmol) was dissolved in dried MeOH (40 mL), and then CH_3_NH_2_ in EtOH (0.31 g, 30%) was added slowly. The mixture was heated to reflux until the starting material had been completely consumed as judged by TLC analysis. The solvent was evaporated under reduced pressure, and the residue was purified by a silica-gel column chromatography (petroleum ether/EtOAc = 3:1) to afford **TMf**.

*(E)-2-(2-(((1-(4-Chlorophenyl)-1*H*-pyrazol-3-yl)oxy)methyl)phenyl)-2-(methoxyimino)-N-methylacetamide* (**TMf**)*.* Yield 90%; Yellow oil; ^1^H-NMR (CDCl_3_) *δ* 7.65 (d, *J* = 2.7 Hz, 1H, CH), 7.58 (d, *J* = 6.9 Hz, 1H, oximino-ArH), 7.51 (d, *J* = 9.0 Hz, 2H, N-ArH), 7.43 (m, 2H, oximino-ArH), 7.37 (d, *J* = 9.0 Hz, 2H, N-ArH), 7.22 (dd, *J* = 1.8, 6.9 Hz, 1H, oximino-ArH), 6.74 (s, 1H, NH), 5.85 (d, *J* = 2.7 Hz, 1H, CH), 5.17 (s, 2H, CH_2_), 3.95 (s, 3H, OCH_3_), 2.89 (d, *J* = 4.8 Hz, 3H, CH_3_). ^13^C-NMR (CDCl_3_) *δ* 163.9, 162.6, 150.8, 138.3, 135.0, 130.1, 129.0, 128.9, 128.3, 128.0, 127.4, 127.3, 127.2, 118.5, 94.2, 68.9, 62.9, 25.9. IR (KBr): IR (KBr): 3406, 3147, 2937, 1682, 1595, 1545, 1484, 1456, 1417, 1358, 1301, 1267, 1237, 1166, 1094, 1027, 981, 932, 884, 830, 783, 751. MS (ESI): calcd for [M+H]^+^ 399.12, [M+Na]^+^ 421.10; found [M+H]^+^ 399.12, [M+Na]^+^ 421.11. Anal. calcd for C_20_H_19_ClN_4_O_3_: C 60.23, H 4.80, N 14.05; found C 60.02, H 4.75, N 14.13.

### 3.3. Fungicidal Activity Assays

Fungicidal activity of **TMa**–**TMf** against *Rhizoctonia solani* was evaluated according to the literature procedures [10,17]. Compounds **TMa**–**TMf** were dissolved in acetone at a concentration of 10 ppm, 1 ppm and 0.1 ppm, respectively, and then added to a sterile agarized Czapek-Dox medium at 45 °C. The control sample contained only one equivalent of acetone. The media were poured onto 8-cm Petri dishes (10 mL for each dish) and after 2 days inoculated with 5-mm PDA discs of overgrown mycelium. The medium was inoculated by a prick of laboratory needle containing fungus spores. The Petri dishes were incubated at r.t. in the dark. After 4 days, the diameters of the inoculation of the cultures were measured. The percentage inhibition of fungal growth was determined by comparison between the development of fungi colonies on media containing compounds and on the control. Three replicates of each test were carried out. Comparative studies involving pyraclostrobin were carried out under the same conditions using solutions in acetone. For the most active compounds, concentrations that would give 90% growth inhibition (EC_90_) were calculated by the statistical method of linear regression between the activity and the logarithm of the concentration.

## 4. Conclusions

In summary, six novel chloro-containing 1-aryl-3-oxypyrazoles **TMa**–**TMf** with an oximino ester or an oximino amide moiety were synthesized by the reaction of 1-aryl-1*H*-pyrazol-3-ols with benzyl bromide. Their fungicidal activity were tested *in vitro* against *Rhizoctonia solani*, and most compounds displayed good fungicidal activity, especially **TMa,** with an optimal EC_90_ lower than 0.1 μg/mL, **TMe** and **TMf** with EC_90_s of 0.20 and 0.32 μg/mL, respectively, which were better than those of pyraclostrobin. In terms of the different positions of chlorine, the sequence of fungicidal activity is Cl-substituted phenyl > Cl-substituted pyrazole. The fungicidal activity was however decreased with increasing chlorine substitution. The present work indicated that **TMa**, **TMe** and **TMf** could be used as potential lead compounds for further studies of novel fungicides.
